# Supplementary material for: Validation of the Emotiv EPOC® EEG gaming system for measuring research quality auditory ERPs
Source: PeerJ. 2013 Feb 19;1:e38. doi: 10.7717/peerj.38 (PMC3628843; doi:10.7717/peerj.38)
Supplement: Table S2 — Right hemisphere descriptive (M and SD) and inferential (t, Cohen’s d, and r) statistics for peak amplitude (mV) and latency (ms) for research (F4) versus gaming (AF4) system comparisons by condition (Passive and Active listening) and tone (Standard and Deviant) for 21 participants. [file peerj-01-38-s002.docx]

| Condition | Tone | ERP | Measure | Research | Gaming | t | Cohen's d |
| --- | --- | --- | --- | --- | --- | --- | --- |
| Passive | Standard | P1 | Amplitude | 0.75 (0.79) | 0.53 (1.42) | -0.74 | 0.19 |
|  |  |  | Latency | 59.71 (14.6) | 63.88 (20.17) | 1.42 | 0.24 |
|  |  | N1 | Amplitude | -3.36 (1.71) | -2.79 (2.34) | 1.18 | 0.29 |
|  |  |  | Latency | 106.29 (8.31) | 111.96 (18) | 1.71 | 0.42 |
|  |  | P2 | Amplitude | 1.87 (1.42) | 1.47 (1.53) | -1.16 | 0.28 |
|  |  |  | Latency | 176.78 (15.69) | 196.91 (35.26) | 3.21 | 0.76 |
|  |  | N2 | Amplitude | -1.34 (1.48) | -1.24 (1.4) | 0.33 | 0.07 |
|  |  |  | Latency | 278.15 (28.16) | 284.73 (41.05) | 0.88 | 0.19 |
|  | Deviant | P1 | Amplitude | 1.3 (1.24) | 1.03 (2.06) | -0.67 | 0.16 |
|  |  |  | Latency | 54.89 (11.02) | 58.6 (17.7) | 0.82 | 0.26 |
|  |  | N1 | Amplitude | -4.43 (2.51) | -3.87 (3.15) | 0.84 | 0.2 |
|  |  |  | Latency | 116.78 (15.7) | 123.94 (24.74) | 1.53 | 0.36 |
|  |  | P2 | Amplitude | 2.29 (1.94) | 2.46 (2.22) | 0.34 | 0.09 |
|  |  |  | Latency | 208.05 (34.43) | 221.93 (42.9) | 1.24 | 0.37 |
|  |  | N2 | Amplitude | -0.71 (1.36) | -1.22 (2.17) | -0.91 | 0.29 |
|  |  |  | Latency | 277.24 (56.45) | 312.74 (60.87) | 2.66 | 0.63 |
| Active | Standard | P1 | Amplitude | 0.99 (0.62) | 0.72 (0.88) | -1.83 | 0.36 |
|  |  |  | Latency | 57.23 (19.94) | 63.16 (18.72) | 1.84 | 0.32 |
|  |  | N1 | Amplitude | -2.78 (1.55) | -2.18 (1.69) | 1.75 | 0.39 |
|  |  |  | Latency | 108.31 (18.27) | 115.8 (18.35) | 2.15 | 0.42 |
|  |  | P2 | Amplitude | 2.35 (1.52) | 1.7 (1.51) | -3.23 | 0.45 |
|  |  |  | Latency | 183.1 (20.78) | 201.4 (29.7) | 3.57 | 0.74 |
|  |  | N2 | Amplitude | -0.71 (1.13) | -0.64 (0.93) | 0.42 | 0.07 |
|  |  |  | Latency | 273.52 (32.95) | 292.8 (27.41) | 2.85 | 0.66 |
|  | Deviant | P1 | Amplitude | 1.17 (1.02) | 1.19 (1.11) | 0.13 | 0.02 |
|  |  |  | Latency | 57.69 (17.33) | 61.86 (19.25) | 0.94 | 0.24 |
|  |  | N1 | Amplitude | -5.29 (1.91) | -4.43 (3.24) | 1.65 | 0.33 |
|  |  |  | Latency | 119.78 (19.27) | 124.01 (19.28) | 0.93 | 0.23 |
|  |  | P2 | Amplitude | 1.57 (2.35) | 2.41 (2.89) | 1.13 | 0.33 |
|  |  |  | Latency | 204.99 (38.9) | 243.29 (69.05) | 2.38 | 0.7 |
|  |  | N2 | Amplitude | -1.54 (2.75) | -1.25 (2.73) | 0.92 | 0.11 |
|  |  |  | Latency | 254.56 (42.15) | 312.87 (84.72) | 2.88 | 0.9 |
